# Nicodemo's method on dental development: a cross-sectional study with 3,271 children and adolescents

**DOI:** 10.1590/1807-3107bor-2024.vol38.0109

**Published:** 2024-11-08

**Authors:** Raquel Porto Alegre Valente, Lorenna Keren Gomes Lima, Juliano Martins Bueno, Millena Barroso Oliveira, Ademir Franco, Luiz Renato Paranhos

**Affiliations:** (a) Universidade Federal de Uberlândia – UFU, School of Dentistry, Uberlândia, MG, Brazil.; (b) Centro Integrado de Radiologia Odontológica, Goiânia, GO, Brazil.; (c) Faculdade São Leopoldo Mandic, School of Dentistry, Department of Forensic Dentistry, Campinas, SP, Brazil.; (d) Universidade Federal de Uberlândia – UFU, School of Dentistry, Department of Preventive and Community Dentistry, Uberlândia, MG, Brazil.

**Keywords:** Statistics as Topic, Forensic Dentistry, Radiography, Tooth

## Abstract

Civil and criminal forensics utilize dental development to estimate age. The method of Nicodemo, Moraes, and Médici Filho (NMM) is a popular dental age estimation tool in South America; however, it lacks a scientific basis for applications in contemporary forensic practice. This research included the largest sample ever collected in Brazil for a similar purpose. The sample consisted of 3,271 panoramic radiographs of female (n = 1,634) and male (n = 1,637) individuals between six and 22.9 years old (mean 14.6 ± 4.9 years). The applied NMM method considered all maxillary and mandibular left permanent teeth (n = 16). The fit between the chronological age and estimated age intervals was assessed, and a correlation test with Lin's correlation coefficient was performed. The overall percentage of fit was 22.5%, without statistically significant differences based on sex (p > 0.05). The percentage of fit was greater in younger individuals, such as those aged 6–6.99 years (90%), and progressively decreased in older individuals, such as those aged 11–11.9 years (18.2%). After 12 years of age, the method could not provide correct classifications up to 25 years of age. Lin's correlation coefficient was predominantly low (ρ = 0.175; 0.367). NMM is considerably limited, and current forensic practice should not apply it to estimate dental age.

## Introduction

In 1974, Nicodemo, Moraes, and Médici Filho (NMM) published a table describing the maturation chronology of permanent teeth.^1^ The original study consisted of the radiographic examination of 478 Brazilian individuals from birth to 25 years of age. The table summarizes three preliminary studies with permanent teeth: a) an investigation of incisors and first molars by Moraes; b) an investigation of canines, premolars, and second molars by Médici Filho;^3^ and c) an investigation of third molars by Nicodemo.^4^ The studies presented the outcomes of each author's doctoral thesis. The table included all permanent teeth classified into eight stages of crown and root formation: I) first evidence of mineralization, II-IV) 1/3, 2/3, and 3/3 of crown formation, V) beginning of root formation, and VI-VIII) 1/3, 2/3, and 3/3 of root formation (the initial names of stages IV and VIII are "complete crown" and "apical closure," respectively).^1^ The table expresses the estimated ages in months and years, depending on the tooth and development stage.^1^ Limitations include broad age intervals and the lack of sex-based age estimates.

Several studies have tested the table,^5-10,^ with the highest prevalence in Brazilian populations. Some illustrated cases of civil^9^ and criminal^10^ legal investigations requiring dental age estimation that applied the NMM method, and others performed original investigations to assess method performance.^5-8^ A strong correlation has been reported between the method's dental development stages and chronological age.^5^ Authors have claimed that this system underestimates chronological age by approximately 0.41 years in women and approximately 0.48 years in men.^6^ However, there is an increasing underestimation tendency as the analyzed individual's age.^6^ That is, the older the individual is, the worse the method performance. A systematic review from 2020, with ten of 2,284 studies, highlighted that Brazilian forensic practice usually applies Nicodemo's method; however, the performance of international methods may be superior.^11^ Another systematic review from 2018 suggested a) moderate use of the method, b) the application of population-specific correction factors, and c) the combination of the NMM with other methods.^12^ Similarly, a systematic review from 2021, with 13 eligible articles of 2,527 and 10 meta-analyzed studies, screened the available scientific literature for dental age estimation methods applied to Brazilian children.^13^ The NMM was not ranked among the best approaches for the Brazilian population.

The compiled state-of-the-art systematic reviews have indicated limitations of the NMM method that should be considered before its use. Hence, the existing scientific gap relies on the possibility of revisiting this method within a sample size that has yet to be addressed in the literature.^12^ This study aimed to revisit the NMM method 50 years after publication to test its performance on 3,271 radiographs from Brazilian children and adolescents.

## Methodology

### Study design and ethical aspects

This observational, analytical, and cross-sectional study was performed in accordance with the Strengthening the Reporting of Observational Studies in Epidemiology (STROBE) guidelines. It complied with the ethics of the Declaration of Helsinki and received approval from the Human Research Ethics Committee of São Leopoldo Mandic College (process number: 49906221.2.0000.5374).

### Setting and data collection

The sample consisted of digital panoramic radiographs taken with an OP300 device (Instrumentarium, Helsinki, Finland) and retrospectively collected from an existing database. The inclusion criteria were as follows: panoramic radiographs of individuals from the central-western region of Brazil, an age interval of 6–22.9 years, and available dates of birth and image acquisition. The exclusion criteria included images of poor quality, bilaterally missing teeth in the dental arch, visible bone and dental lesions, visible evidence of maxillofacial deformities, and surgical or orthopedic appliances.

**Figure 1:**
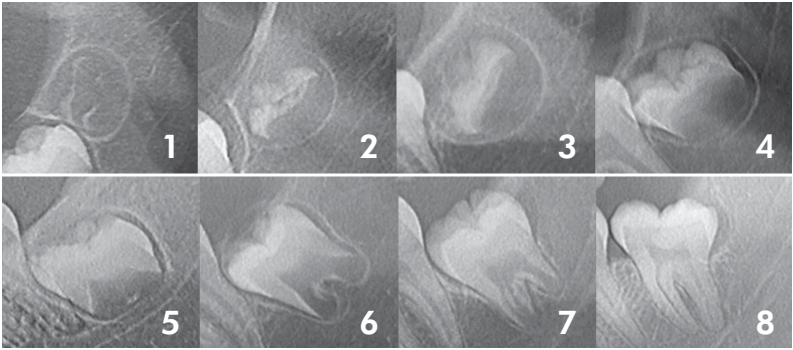
Eight dental development stages were proposed by Nicodemo et al.^1^: 1) first evidence of mineralization, 2) 1/3 of crown formation, 3) 2/3 of crown formation, 4) crown completion, 5) beginning of root formation, 6) 1/3 of root formation, 7) 2/3 of root formation, and 8) apical closure.

**Figure 2:**
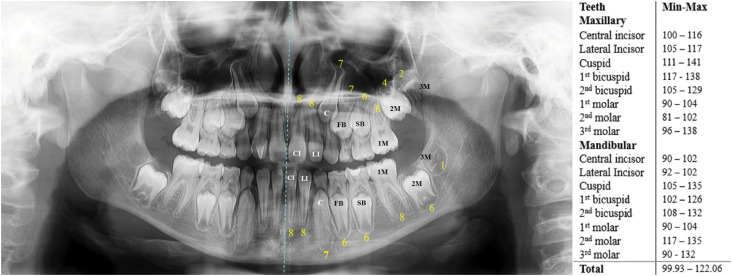
Illustrative examples of the classification of maxillary and mandibular left central incisors (CIs), lateral incisors (LIs), canines (C), first premolars/bicuspids (FBs), second premolars/bicuspids (SBs), and first (1 M), second (2 M), and third (3 M) molars. The estimated age interval is presented alongside and was calculated via the means of the minimal (Min) and maximum (Max) dental age limits, expressed in months.

A trained dentist analyzed the radiographs with an image viewer. The anonymized analysis allowed the reduction (zooming out) and magnification (zooming in) of images and adjustments in brightness and contrast. The analyzers were blinded to the patient's age and sex and performed in a dimmed room. No more than 50 radiographs were analyzed daily to avoid visual fatigue. The eight-stage system (Figure 1) proposed by Nicodemo et al.^1^ classified all left permanent teeth (quadrants #2 and #3), including third molars. Age was estimated by converting tooth stages into minimum and maximum age intervals via the original table of the NMM for the chronological development of permanent teeth^1^ (Figure 2). The estimated age was recorded for each tooth. Hence, the same individual would have 16 values representing the minimum age and 16 representing the maximum age within the estimated age intervals.

The chronological age was calculated for all individuals to enable comparisons with the estimated age. The calculation considers the difference between the date of radiographic image acquisition and the individual's date of birth. Age was then converted from a continuous numeric variable into a categorical variable. More specifically, seventeen age groups were established from six to 22.9 years. Each group had an interval of one year (*i.e.*, 6–6.99 years, 7–7.99 years, etc.). The number of individuals within each group was balanced (approximately 6% of the sample, or 197 individuals similarly distributed between females and males per age group).

The study recruited a second trained researcher to enable the interobserver reproducibility test, and the primary researcher revisited the sample after the first analysis to allow the intraobserver reproducibility test. The intra- and interobserver analyses used 100 radiographs (1,600 tooth positions) and were performed 30 days after the primary analysis. Weighted kappa was used to compare the staging from the researchers’ analyses and yielded reproducibility values above 0.8. A third researcher with 13 years of experience in forensic dentistry supervised the analyses.

### Data analysis

The data were initially explored via descriptive statistics. The means and standard deviations (SDs) of the minimum and maximum values per age interval from the original NMM table were calculated for the present sample. Lin's concordance coefficient was used to assess the agreement between estimated and chronological ages. This assessment was performed based on sex and age group for the total sample and individually. Additionally, the absolute and relative numbers of radiographs with a chronological age fitting the estimated age interval were calculated based on sex and age group. Fisher's exact test was used to investigate the differences between sexes. The following equation was used to calculate the error of the method (expressed in years): Difference = estimated age – chronological age. All analyses considered a 5% statistical significance and a 95% confidence interval. The study used Stata software v.18.0 (StataCorp LLC, College Station, USA).

## Results

The sample consisted of 3,271 panoramic radiographs of females (n = 1634) and males (n = 1637) between the ages of six and 22.9 years (mean 14.6 ± 4.9 years) (Table 1).

**Table 1:** Sample distribution based on sex and age group.

Variable		Total sample		Female		Male	
		n	%	n	%	n	%
Sex							
	Female	1634	50.0				
	Male	1637	50.0				
Age group (years)							
	6.0–6.9	192	5.9	97	5.9	95	5.8
	7.0–7.9	185	5.7	94	5.8	91	5.6
	8.0–8.9	178	5.4	91	5.6	87	5.3
	9.0–9.9	177	5.4	89	5.5	88	5.4
	10.0–10.9	175	5.4	87	5.3	88	5.4
	11.0–11.9	187	5.7	88	5.4	99	6.0
	12.0–12.9	200	6.1	100	6.1	100	6.1
	13.0–13.9	200	6.1	100	6.1	100	6.1
	14.0–14.9	198	6.1	99	6.1	99	6.0
	15.0–15.9	198	6.1	99	6.1	99	6.0
	16.0–16.9	189	5.8	91	5.6	98	6.0
	17.0–17.9	199	6.1	99	6.1	100	6.1
	18.0–18.9	199	6.1	100	6.1	99	6.0
	19.0–19.9	197	6.0	100	6.1	97	5.9
	20.0–20.9	197	6.0	98	6.0	99	6.0
	21.0–21.9	199	6.1	100	6.1	99	6.0
	22.0–22.9	198	6.0	99	6.1	99	6.0
		Mean	SD	Mean	SD	Mean	SD
Age (years)		14.6	4.9	14.6	4.9	14.6	4.9

n: absolute number of radiographs; SD: standard deviation; .

The means calculated for the minimum values were between 7.3 (SD = 0.5) years, observed in the maxillary first molar (tooth #26), and 12.7 (SD = 5.1) years, observed in the maxillary and mandibular third molars (teeth #28 and #38). The means calculated for the maximum values were between 8.4 (SD = 0.3) years, observed in mandibular central and lateral incisors (teeth #31 and #32), and 15.7 (SD = 5.7) years, observed in teeth #28 and #38 (Table 2).

**Table 2:** Means and standard deviations of the minimum and maximum ages for each tooth.

Tooth (FDI)	Minimum value					Maximum value			
	n	Mean	SD	Min	Max	Mean	SD	Min	Max
21	3275	8.1	0.6	3.0	8.3	9.5	0.6	4.8	9.7
22	3274	8.4	0.8	2.8	8.8	9.5	0.6	4.8	9.8
23	3275	9.6	1.4	5.0	10.5	11.9	1.8	6.5	13.0
24	3275	9.8	1.5	4.8	10.8	12.0	1.9	6.3	13.3
25	3273	10.4	1.9	4.3	14.8	12.1	1.7	5.5	14.8
26	3275	7.3	0.5	3.0	7.5	8.6	0.4	4.0	10.3
27	3274	11.0	2.2	3.3	12.5	12.3	1.9	4.8	13.5
28	2918	12.7	5.1	0.0	18.0	15.7	5.7	0.0	20.4
38	2973	12.7	5.1	0.0	18.0	15.7	5.5	0.0	20.4
37	3275	11.0	2.1	4.3	12.5	12.5	2.0	5.5	13.8
36	3275	7.4	0.4	1.5	7.5	8.6	0.3	3.8	8.7
35	3274	10.5	1.8	2.8	11.8	12.2	1.7	4.6	13.3
34	3275	9.9	1.8	4.3	11.0	11.9	1.8	6.0	13.0
33	3275	9.7	1.7	2.0	11.0	11.9	1.8	4.5	13.0
32	3274	7.4	0.6	1.5	7.7	8.4	0.3	5.5	16.8
31	3101	7.4	0.4	4.0	7.5	8.4	0.2	5.7	8.5

FDI: International Dental Federation; n: absolute number of analyzed teeth; SD: standard deviation; Min: minimum; Max: maximum. Age expressed in years.

For the total sample, the percentage of fit of the chronological age within the estimated age intervals between the minimum and maximum ages was 22.5% (Table 3). This percentage did not differ between females and males within the age intervals (p = 0.081) and decreased from younger to older individuals (Table 3). By the age of 6–6.99 years, approximately 90% of the chronological ages fit within the estimated age intervals, progressively decreasing to 18.2% in the 11–11.9 years age group. The fit between chronological and estimated ages did not occur after 12 years. The only significant difference based on sex was detected in the 9–-9.99 year age group, in which females had a greater percentage of fit (Table 3).

**Table 3:** Percentages of fit between the chronological age and estimated age intervals for females and males and per age group.

Variable		Total sample			Female			Male			p-value*
		n	%	95%CI	n	%	95%CI	n	%	95%CI	
Combined teeth											0.081
	Outside the interval	2538	77.5	76.0–78.9	1245	76.2	74.1–78.2	1289	78.7	76.7–80.7	
	Within the interval	737	22.5	21.1–24.0	389	23.8	21.8–25.9	348	21.3	19.3–23.3	
Age group^a^											
	6.0–6.9	173	90.1	85.0–93.6	86	88.7	80.7–93.6	87	91.6	84.0–95.7	0.630
	7.0–7.9	151	81.6	75.4–86.6	79	84.0	75.2–90.1	72	79.1	69.6–86.3	0.450
	8.0–8.9	128	71.9	64.9–78.0	70	76.9	67.2–84.4	58	66.7	56.1–75.8	0.137
	9.0–9.9	137	77.4	70.7–83.0	79	88.8	80.4–93.8	58	65.9	55.4–75.0	< 0.001
	10.0–10.9	114	65.1	57.8–71.8	57	65.5	55.0–74.7	57	64.8	54.3–74.0	1.000
	11.0–11.9	34	18.2	13.3–24.4	18	20.5	13.3–30.2	16	16.2	10.1–24.8	0.455
	12.0–12.9	0	0.0	-	0	0.0	-	0	0.0	-	-
	13.0–13.9	0	0.0	-	0	0.0	-	0	0.0	-	-
	14.0–14.9	0	0.0	-	0	0.0	-	0	0.0	-	-
	15.0–15.9	0	0.0	-	0	0.0	-	0	0.0	-	-
	16.0–16.9	0	0.0	-	0	0.0	-	0	0.0	-	-
	17.0–17.9	0	0.0	-	0	0.0	-	0	0.0	-	-
	18.0–18.9	0	0.0	-	0	0.0	-	0	0.0	-	-
	19.0–19.9	0	0.0	-	0	0.0	-	0	0.0	-	-
	20.0–20.9	0	0.0	-	0	0.0	-	0	0.0	-	-
	21.0–21.9	0	0.0	-	0	0.0	-	0	0.0	-	-
	22.0–22.9	0	0.0	-	0	0.0	-	0	0.0	-	-

* p-value after Fisher's exact test for heterogeneity between females and males

a Frequencies of chronological ages that fit within the age group (interval); age expressed in years; n: the number of occurrences; CI: confidence interval.

A new analysis excluded third molars from the interval calculations to prevent the interaction of these teeth from biasing the age intervals. The outcomes were similar to those of the analysis of all teeth. The primary difference was the fit reduction in the age intervals of 6–-6.99, 10–-10.99, and 11–-11.99 years (Table 4). Lin's concordance coefficient was greater when all teeth (including the third molars) were combined. However, all analyses per age group revealed moderate to low concordance (between 0.175 and 0.367) (Table 5).

**Table 4:** Percentages of fit between the chronological age and estimated age intervals for females and males and per age group, excluding third molars.

Variable		Total sample			Female			Male			p-value*
		n	%	95%CI	n	%	95%CI	n	%	95%CI	
Combined teeth, except third molars											0.116
	Outside the interval	2585	78.9	77.5–80.3	1271	77.8	75.7–79.7	1310	80.0	78.0–81.9	
	Within the interval	690	21.1	19.7–22.5	363	22.2	20.3–24.3	327	20.0	18.–22.0	
Age group^a^											
	6.0–6.9	140	72.9	66.2–78.7	68	70.1	60.3–78.4	72	75.8	66.2–83.4	0.419
	7.0–7.9	166	89.7	84.5–93.4	83	88.3	80.1–93.4	83	91.2	83.4–95.5	0.630
	8.0–8.9	155	87.1	81.3–91.3	82	90.1	82.1–94.8	73	83.9	74.6–90.2	0.266
	9.0–9.9	145	81.9	75.5–86.9	82	92.1	84.4–96.2	63	71.6	61.3–80.0	< 0.001
	10.0–10.9	77	44.0	36.8–51.4	44	50.6	40.2–60.9	33	37.5	28.0–48.0	0.095
	11.0–11.9	7	3.7	1.8–7.6	4	4.6	1.7–11.5	3	3.0	1.0–8.9	0.708
	12.0–12.9	0	0.0	-	0	0.0	-	0	0.0	-	-
	13.0–13.9	0	0.0	-	0	0.0	-	0	0.0	-	-
	14.0–14.9	0	0.0	-	0	0.0	-	0	0.0	-	-
	15.0–15.9	0	0.0	-	0	0.0	-	0	0.0	-	-
	16.0–16.9	0	0.0	-	0	0.0	-	0	0.0	-	-
	17.0–17.9	0	0.0	-	0	0.0	-	0	0.0	-	-
	18.0–18.9	0	0.0	-	0	0.0	-	0	0.0	-	-
	19.0–19.9	0	0.0	-	0	0.0	-	0	0.0	-	-
	20.0–20.9	0	0.0	-	0	0.0	-	0	0.0	-	-
	21.0–21.9	0	0.0	-	0	0.0	-	0	0.0	-	-
	22.0–22.9	0	0.0	-	0	0.0	-	0	0.0	-	-

* p-value after Fisher's exact test for heterogeneity between females and males

a Frequencies of chronological ages that fit within the age group (interval); age expressed in years; n: the number of occurrences; CI: confidence interval.

**Table 5:** Differences between estimated and chronological ages and Lin's concordance coefficient between them.

Variable		n	Mean age		Difference between estimated and chronological ages		Lin's coefficient	
			Chronological	Estimated	Difference	SD	p-value	95%CI
Combined teeth								
	Mean of the minimum	3270	14.6	9.5	-5.1	3.5	0.269	0.259–0.279
	Mean of the maximum	3270	14.6	11.2	-3.4	3.6	0.367	0.355–0.379
Excluding third molars								
	Mean of the minimum	3270	14.6	9.1	-5.5	4.0	0.175	0.167–0.183
	Mean of the maximum	3270	14.6	10.7	-3.9	4.0	0.223	0.213–0.232

Age is expressed in years; n: the number of radiographs; SD: standard deviation; CI: confidence interval.

The analyses per age group revealed the same tendency for low correlation coefficients and a lack of statistically significant differences between females and males (p > 0.05) when all teeth (Table 6) and excluding third molars (Table 7) were combined.

**Table 6:** Differences between estimated and chronological ages and Lin's concordance coefficient per age group and all teeth.

Variable		n	Mean age			Mean difference		Lin's coefficient for the minimum age		Lin's coefficient for the maximum age	
			Chronological	Estimated minimum	Estimated maximum	Minimum	Maximum	p-value	95%CI	p-value	95%CI
Sex											
	Female	1634	14.6	9.5	11.3	-5.1	1.8	0.263	0.249–0.277	0.359	0.342–0.375
	Male	1637	14.6	9.5	11.2	-5.1	1.7	0.276	0.261–0.290	0.375	0.359–0.392
Age group											
	6.0–6.9	192	6.5	5.9	7.4	-0.7	0.9	0.139	0.086–0.192	0.100	0.061–0.139
	7.0–7.9	185	7.5	6.5	8.2	-1.0	0.7	0.106	0.069–0.143	0.158	0.101–0.215
	8.0–8.9	178	8.5	7.3	9.0	-1.2	0.5	0.085	0.055–0.115	0.203	0.136–0.269
	9.0–9.9	177	9.5	8.2	10.0	-1.3	0.5	0.036	0.013–0.059	0.111	0.036–0.185
	10.0–10.9	175	10.5	8.8	10.6	-1.7	0.1	0.033	0.019–0.047	0.268	0.158–0.372
	11.0–11.9	187	11.5	9.2	11.1	-2.3	-0.4	0.008	0.002–0.014	0.105	0.024–0.183
	12.0–12.9	200	12.5	9.2	11.1	-3.2	-1.3	0.004	0.001–0.007	0.019	0.004–0.035
	13.0–13.9	200	13.5	10.0	11.7	-3.5	-1.7	0.003	0.001–0.006	0.009	0.002–0.016
	14.0–14.9	198	14.5	10.3	12.0	-4.2	-2.5	0.002	0.001–0.003	0.005	0.002–0.008
	15.0–15.9	198	15.5	10.4	12.1	-5.0	-3.3	0.001	0.000–0.002	0.002	0.001–0.004
	16.0–16.9	189	16.5	10.5	12.1	-6.1	-4.4	0.000	0.000–0.001	0.001	-0.001–0.003
	17.0–17.9	199	17.5	10.7	12.4	-6.8	-5.1	0.000	0.000–0.000	0.000	-0.001–0.001
	18.0–18.9	199	18.5	10.8	12.5	-7.7	-6.0	0.000	0.000–0.000	0.000	0.000–0.000
	19.0–19.9	197	19.5	10.8	12.4	-8.8	-7.1	0.000	0.000–0.000	0.000	0.000–0.000
	20.0–20.9	197	20.5	10.9	12.5	-9.6	-8.0	0.000	0.000–0.000	0.000	0.000–0.000
	21.0–21.9	199	21.5	10.9	12.6	-10.6	-8.9	0.000	0.000–0.000	0.000	0.000–0.000
	22.0–22.9	198	22.5	10.9	12.6	-11.5	-9.9	0.000	0.000–0.000	0.000	0.000–0.000

Age is expressed in years; n: the number of radiographs; CI: confidence interval.

**Table 7:** Difference between estimated and chronological ages and Lin's coefficient of concordance excluding third molars.

Variables		n	Mean age			Mean difference		Lin's coefficent for the minimum age		Lin's coefficent for the maximum age	
			Chronological	Estimated minimum	Estimated maximum	Minimum	Maximum	p-value	95%CI	p-value	95%CI
Sex											
	Females	1634	14.6	9.2	10.7	-5.4	1.5	0.170	0.159–0.180	0.215	0.202–0.227
	Males	1637	14.6	9.1	10.7	-5.5	1.6	0.180	0.169–0.192	0.230	0.217–0.243
Age category											
	6.0–6.9	192	6.5	6.2	7.8	-0.3	1.3	0.233	0.143–0.320	0.056	0.034–0.079
	7.0–7.9	185	7.5	6.8	8.5	-0.7	1.0	0.163	0.108–0.218	0.090	0.056–0.123
	8.0–8.9	178	8.5	7.5	9.2	-1.0	0.7	0.103	0.065–0.140	0.145	0.091–0.198
	9.0–9.9	177	9.5	8.3	9.9	-1.2	0.4	0.048	0.024–0.072	0.171	0.091–0.250
	10.0–10.9	175	10.5	8.8	10.4	-1.7	0.0	0.031	0.018–0.044	0.342	0.215–0.458
	11.0–11.9	187	11.5	9.1	10.8	-2.4	-0.7	0.006	0.002–0.011	0.049	0.012–0.087
	12.0–12.9	200	12.5	9.2	10.8	-3.3	-1.6	0.003	0.001–0.006	0.012	0.003–0.021
	13.0–13.9	200	13.5	9.7	11.3	-3.8	-2.2	0.002	0.000–0.003	0.003	0.001–0.006
	14.0–14.9	198	14.5	9.9	11.4	-4.6	-3.1	0.000	0.000–0.001	0.000	0.000–0.001
	15.0–15.9	198	15.5	9.9	11.4	-5.6	-4.1	0.000	0.000–0.000	0.000	0.000–0.000
	16.0–16.9	189	16.5	9.8	11.3	-6.7	-5.2	0.000	0.000–0.000	0.000	0.000–0.001
	17.0–17.9	199	17.5	9.9	11.4	-7.6	-6.0	0.000	0.000–0.000	0.000	0.000–0.000
	18.0–18.9	199	18.5	9.9	11.4	-8.6	-7.1			0.000	0.000–0.000
	19.0–19.9	197	19.5	9.9	11.4	-9.6	-8.1	0.000	0.000; 0.000		
	20.0–20.9	197	20.5	9.9	11.4	-10.6	-9.1				
	21.0–21.9	199	21.5	10.0	11.5	-11.5	-10.0	0.000	0.000; 0.000	0.000	0.000; 0.000
	22.0–22.9	198	22.5	10.0	11.5	-12.5	-11.0	0.000	0.000; 0.000	0.000	0.000; 0.000

Age expressed in years; n: number of radiographs; CI: confidence interval.

## Discussion

What makes a dental age estimation method popular? Answers committed to science should include terms such as "accuracy," "populational validity," "methodological soundness," and "viability." This might be true for some approaches employed in the XXI century, such as Willems’^14^ and Cameriere's^15,16^ methods and the London Atlas.^17^ Some older techniques, such as Demirjian's^18^ method, have probably become prevalent because of the scarcity of statistically developed methods and the large sample sizes, a turning point in scientific methods for dental age estimation. The NMM method might not be well known abroad, but it is among the most popular methods of aging in Brazil. The authors claim that the system is user-friendly and does not require previous examiner experience^5^ (this statement should be carefully approached, if not entirely avoided, in the forensic context). The method allows age estimation from any permanent teeth. However, the NMM is merely a table reflecting the mean ages of certain groups of individuals per tooth development stage. For example, it could serve as a reference auxiliary table for simple clinical investigations of the biological development of children. Conversely, the method became popular for forensic purposes, a field whose consequences might harm the present and future of examined individuals.

Initially, the NMM was a cross-sectional depiction of the studied population, expressed in age intervals until 25. A critical analysis of this method allows quick observation of limitations that may jeopardize forensic practice. First, the staging system used by the authors is considerably scarce (describing only eight stages). This promotes large "jumps" between stages, providing large original age intervals. The tooth development process might be more detailed with more stages. For example, Moorrees’ system^19^ has been endorsed and applied in other approaches, such as the London Atlas^17^ and Anderson's^20^ methods. Combined with this limitation, a sample size of less than 500 individuals is too small for the broad 0–-25-year age interval claimed by NMM's authors. If individuals are evenly distributed based on sex and age (one year for each group, which has recently become common),^21-23^ fewer than ten females and ten males would be expected per age group. This does not seem representative enough, mainly because this method remains applicable to newer generations of forensically examined individuals.

Forensic experts must consider that the NMM method, published in 1974, uses radiographs from individuals born much earlier. For example, the oldest individuals in the sample were 25 years old. If a 25-year-old's radiograph is taken in 1974, which is unlikely, that person would have been born in 1949. This scenario suggests a potentially significant generational change, given the different habits of younger individuals to whom the NMM method has been more recently applied.

The sample in this study included individuals from the central-western region of Brazil. The rationale behind this specific geographic source of images was based on the shortage of previous studies in this region. Unlike those in the southeastern region, the forensic teams in central-western Brazil do not have various decision-making analyses to support their routine with evidence-based findings. Hence, this research aimed to support central-west services by testing the NMM, one of the dental age estimation tools most commonly used in Brazil. Our outcomes showed that the best fit between chronological age and estimated age intervals was within the youngest age groups, with a percentage of fit of up to 90%. Authors,^24^ who analyzed a sample from the Southeast Region, reported rates between 70% and 90% in individuals between seven and 11 years old. From 13 years onward, no fit was detected between chronological age and estimated age intervals. The same occurred in our sample from the age of 12 onward. The small differences between studies might have emerged because we analyzed 16 tooth positions per individual, whereas the other authors analyzed five. Moreover, our sample was ten times larger than the authors’ sample.

Corroborating our findings, a previous study that analyzed 16 teeth (eight maxillary teeth and eight mandibular teeth) revealed the worst percentage of fit among individuals older than 15.8%, which occurred because of the lower degree of dental development in older adolescents and young adults than children. Conversely, the authors found higher percentages of fit when only four teeth were used to estimate age instead of 16 teeth. This may contradict the improved age estimation in the presence of more development structures but could be explained by the challenges of radiographic visualization. Adding teeth to the analysis will jeopardize age estimation outcomes if the staging procedure is unclear, as evidenced by subsequent findings. When teeth are used individually (without combining tooth positions), the authors reported a greater percentage of fit in third molars, indicating that third molars appear optimal for estimating age in the studied age interval (10–25 years).^8^ Their conclusions should be carefully considered because third molars’ developmental variability might hamper age estimation. Currently, these teeth should be considered only as the last resort to obtain development information for age estimation,^25^ usually after 16 years. The authors found utility in third molars because of their more distinct visualization in the mandible than other teeth (and tooth apices).

This study did not detect differences in the percentage of fit between the chronological age and estimated age intervals based on sex. Other authors confirmed this finding.^8,24^ The original NMM table did not distinguish females and males, but the influence of sex on dental development and age estimation is currently more addressed. Hence, researchers and forensic experts must consider the possible application of sex-specific methods in their routines. A systematic review and meta-analysis from 2021 listed and ranked the best radiographic methods for estimating the dental age of Brazilian children.^13^ The authors supported Willems's^14^ and Cameriere's^15^ methods, which were validated in different Brazilian samples, based on the mineralization process of permanent teeth; hence, these methods are suitable for individuals younger than 15 years. Another method is the London Atlas,^17^ which has been revisited worldwide and has demonstrated proper applicability for the dental age estimation of children, including Brazilians. The NMM could be replaced with newer, internationally tested^11^ and accepted methods concerning adolescents and young adults, such as the London Atlas^17^ and Cameriere's.^15,16^ These systems, applied to children and adolescents, are superior to the NMM in reducing the differences between chronological and estimated ages and are endorsed for optimal transitions of the Brazilian forensic routine toward more evidence-based practice.

## Conclusion

Fifty years after its publication, the NMM method requires a recall. This study tested the percentage of fit of the NMM method in the largest sample ever collected, revealing that two individuals from every ten analyzed had their age correctly classified. Moreover, the NMM method should not be applied to individuals older than 12. In practice, this approach should not be used for forensics, especially when concerning the transition between childhood and adolescence (legal age of 12 years), sexual consent (legal age of 14 years), relative ability (legal age of 16 years), and civil/criminal majority (legal age of 18 years).
